# Erectile Dysfunction in Relation to Metabolic Disorders and the Concentration of Sex Hormones in Aging Men

**DOI:** 10.3390/ijerph19137576

**Published:** 2022-06-21

**Authors:** Rył Aleksandra, Szylińska Aleksandra, Rotter Iwona

**Affiliations:** Department of Medical Rehabilitation and Clinical Physiotherapy, Pomeranian Medical University, 70-204 Szczecin, Poland; aleksandra.szylinska@pum.edu.pl (S.A.); iwona.rotter@pum.edu.pl (R.I.)

**Keywords:** erectile dysfunctions, metabolic disorders, testosterone, body fat, aging men

## Abstract

Background: The aim of this study was to analyze the relationship between the prevalence of erectile dysfunction in men as diagnosed by the International Index of Erectile Function (IIEF) questionnaire and the respective levels of sex hormones and biochemical parameters, as well as indices of visceral fat accumulation and activity. Material and Methods: The study comprised 148 male (60–75 years) patients from primary care outpatient clinics in the city of Szczecin (Poland). The men were asked to complete a shortened survey questionnaire with sociodemographic data, as well as a shortened version of the IIEF (five items). Venous blood samples were collected. Total testosterone (TT), estradiol (E2), sex hormone-binding globulin (SHBG), dehydroepiandrosterone sulfate (DHEAS), total cholesterol (ChT), low-density lipoprotein (LDL), high-density lipoprotein (HDL), triglycerides (TG), fasting plasma glucose (FPG) and albumin were determined. Lipid accumulation product (LAP) and visceral adiposity index (VAI) were calculated. Results: A correlation was found in the analysis of LAP index values (OR = 1.017; *p* = 0.050). The analysis of hormone concentrations showed a correlation between the diagnosed trait and the value of TT (OR = 1.216; *p* = 0.046) and SHBG (OR = 1.020; *p* = 0.007). Conclusions: VAI and LAP have been shown to be good indicators for assessing erectile dysfunction in men over 60 years of age.

## 1. Introduction

Research into male sexual health is a relatively neglected field of medical science. This is due to several factors such as low rates of patients seeking treatment due to a sense of shame and embarrassment and their reluctance to participate in such research. Epidemiological data show that the problem is significant, with a high prevalence of erectile dysfunction among men worldwide (19.2%, with a steep age-related increase, 2.3–53.4%). [1,2,3]—mainly erectile dysfunction (ED) and premature ejaculation (PE). Erection is a complex phenomenon that is governed by a balance between neurological, vascular, and tissue regulation and is associated with arterial dilation, trabecular smooth muscle, relaxation, and activation of the corporeal veno-occlusive mechanism [4].

According to the Fourth International Consultation on Sexual Medicine, ED is a persistent or recurrent inability to achieve and/or maintain a penile erection to permit satisfactory sexual activity [5].

Another problem is premature ejaculation, which, according to the 2013 guidelines by the International Society for Sexual Medicine (ISSM) Ad Hoc, occurs within about 1 minute of vaginal penetration as the first sexual event, or as a clinically significant reduction in latency time to about 3 min or less and the inability to delay ejaculation in all or nearly all vaginal penetrations, with negative emotional consequences, such as distress, bother, frustration and/or the avoidance of sexual intimacy [6]. Testosterone is considered the principal hormone involved in male gonad formation and ejaculation control. However, there are contradictory data regarding the correlation between testosterone levels and PE [7,8].

One available tool that examines ED is the International Index of Erectile Function (IIEF)—a widely used multidimensional instrument for assessing male sexual function. It is recommended as a baseline for ED testing and is recommended for assessing the severity of erectile dysfunction [9]. Erectile dysfunction affects the physical and psychosocial health of both men and their partners, which can then have an adverse impact on their quality of life. In addition, there are reports that ED may be an early symptom of peripheral vascular and coronary artery disease, and so erectile dysfunction may be related to cardiovascular disease (CVD) [10,11].

Analyzing ED-related data in aging men in the context of their metabolic disorders is a new perspective on this problem. Given the age-related changes in hormone concentrations and the problem of a large proportion of obese patients, these problems should be considered comorbid. The aim of this study was to analyze the relationship between the prevalence of erectile dysfunction in men as diagnosed by the IIEF questionnaire and the respective levels of sex hormones and biochemical parameters, as well as indices of visceral fat accumulation and activity.

## 2. Materials and Methods

### 2.1. Study Participants

The study comprised 148 male patients from primary care outpatient clinics in the city of Szczecin (Poland). After a preliminary analysis of questionnaires, it was found that 42 (28.3%) of the men from the group had declared a lack of sexual activity over the previous 4 weeks, so they were excluded from the results. Other exclusion criteria for the study were a history of cancer, alcohol abuse, liver or kidney failure, diabetes, New York Heart Association (NYHA) class III or IV heart failure, or use of the medications—neuroleptics, chemotherapeutic agents, immunosuppressants, corticosteroids, and antidepressants. A final number of 106 men aged 60 to 75 years completed the study.

The men were asked to complete a shortened survey questionnaire with sociodemographic data, as well as a shortened version of the International Index of Erectile Function (IIEF) (five items). This questionnaire is designed for self-assessment of sexual function over the past 4 weeks. It enables differentiation of erectile dysfunction in the areas of erectile dysfunction, reaching orgasm, sexual desire, as well as satisfaction with sexual life and overall sexual satisfaction [12]. A score of 21 points was recognized as the qualification threshold. A value of 21 points or more was the qualification for the group with erectile dysfunction, and a value above was the qualification for the group without erectile dysfunction.

### 2.2. Determination of Sex Hormones

Venous blood samples were collected from the elbow vein on an empty stomach between 07:00 and 10:00 am. The samples were stored at −20 °C. Determinations were performed by ELISA (DRG Medtek, Warsaw, Poland). Total testosterone (TT, normal range for men: 2.36–9.96 ng/mL), estradiol (E2, normal range for men: 11.2–50.4 pg/mL), sex hormone-binding globulin (SHBG, normal range for men: 18–110 nmol/L), and dehydroepiandrosterone sulfate (DHEAS, normal range for men: 110–470 µg/dL) were determined. Free testosterone (FT, normal range for men: 8.9–45.5 pg/mL) was calculated using the formula developed by Vermeulen: FT = ((TT − N − SHBG + √ ((N + SHBG − TT)2 + 4 × N × TT))/2N)) × (100/TT), where N = 0.5217 × albumin concentration + 1 [13]. The bioT concentration was calculated using the following formula: bioT = FT concentration (ng/dL) + albumin concentration [14].

The sensitivity of the methods for total testosterone was 0.083 ng/mL with a limit of quantification (LOQ) of 0.249 ng/mL; the intra-assay and inter-assay coefficients of variation (CV) were 3.28% and 6.71%, respectively. The sensitivity of the SHBG assay was 0.001 nmol/L with an LOQ of 1.65 nmol/L; the inter-assay and intra-assay CVs were 3.0% and 7.2%, respectively. E2 test sensitivity was 9.714 pg/mL with an LOQ of 29.142 pg/mL; inter- and intra-test CVs were 6.72% and 2.71%. The DHEAS CV sensitivity score between tests was 8.9%, and the CV within the test was 10.5%.

The homeostatic model assessment for insulin resistance (HOMA-IR) value was calculated using the formula: (fasting blood insulin concentration (mu/mL) × fasting blood glucose concentration (mmol/L))/22.5. The lipid accumulation product (LAP) index as a predictor of the risk of diabetes and cardiovascular disease was calculated using the formula ((waist circumference (cm) − 65) × TG (mmol)) [15]. The visceral adiposity index (VAI) was calculated to assess visceral fat accumulation and cardiometabolic risk using the formula: VAI = waist circumference (cm)/(39.68 + (1.88 × BMI)) × ((TG/1.03) × (1.31/HDL)) [16].

### 2.3. Determination of the Concentrations of Biochemical Parameters

Total cholesterol (ChT), low-density lipoprotein (LDL), high-density lipoprotein (HDL), triglycerides (TG), fasting plasma glucose (FPG), and albumin were determined using the standard methods in a diagnostic laboratory (all parameters were determined using direct methods).

### 2.4. Statistical Analysis

Statistical analysis was performed using Statistica v13 (StatSoft Poland, Cracow, Poland). Quantitative variables were presented as means, minimums, maximums, and standard deviations (SD). Data were checked for normality using a Shapiro–Wilk test. In the case of a normal distribution, means were compared using a Student’s t-test; otherwise, the nonparametric Mann–Whitney U test was used. Multiple logistic regression models were adjusted for age. A receiver operating characteristic (ROC with the Youden index) curve analysis was used to assess the accuracy of the TT, SHBG, VAI, and LAP. The significance level was set at p ≤ 0.05.

## 3. Results

Scores from the 106 participating men were used in the data analysis. Based on the IIEF questionnaire, 72 men scored less than 21 on the questionnaire (patients with erectile dysfunction), and 34 men scored above 21 (patients without erectile dysfunction). Table 1 details the analysis of the relationship between groups with anthropometric parameters and indices describing body weight and lipid accumulation depending on the score obtained in the questionnaire. It was shown that men in both groups differed in the analysis of waist–hip ratio (WHR) (*p* = 0.044), VAI index value (*p* = 0.048), and LAP index value (*p* = 0.050).

The relationship between hormonal (Table 2) and biochemical (Table 3) parameters in the men showed that those with erectile dysfunction had statistically significantly lower TT concentration (*p* = 0.018), SHBG concentration (*p* = 0.026), and albumin concentration (*p* = 0.029) than those without dysfunction. There were no other significant differences in the concentrations of other biochemical parameters between the two groups.

Multivariate logistic regression analysis was also performed for the International Index of Erectile Function scores adjusted for age (Table 4). A correlation was found in the analysis of LAP index values (OR = 1.017; *p* = 0.050). The analysis of hormone concentrations showed a correlation between the diagnosed trait and the value of TT (OR = 1.216; *p* = 0.046) and SHBG (OR = 1.020; *p* = 0.007).

**Figure 1:**
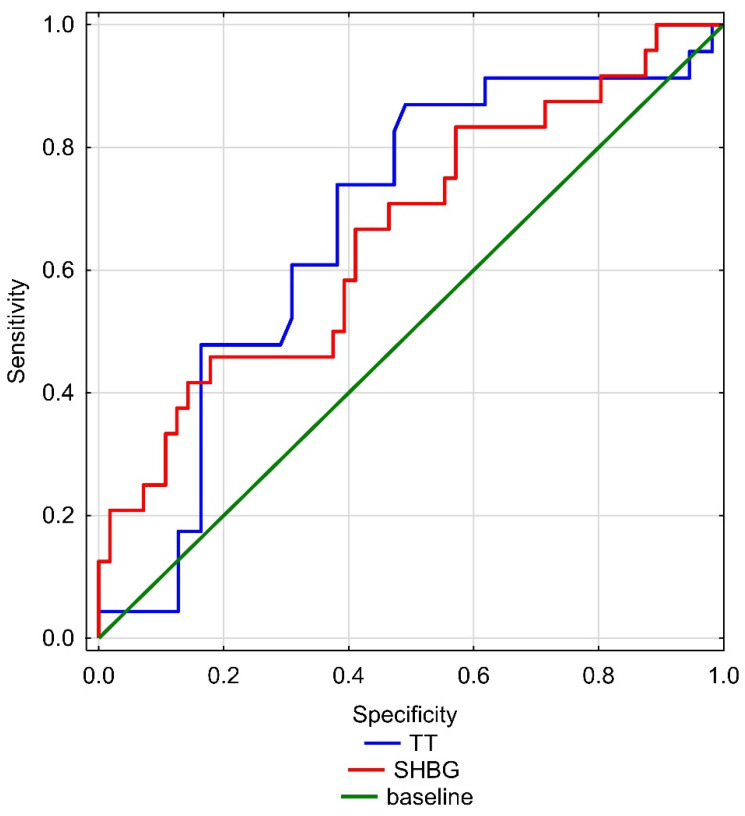
Receiver operating characteristics of the concentrations of TT (blue line) and SGBH (red line) for the International Index of Erectile Function.

**Figure 2:**
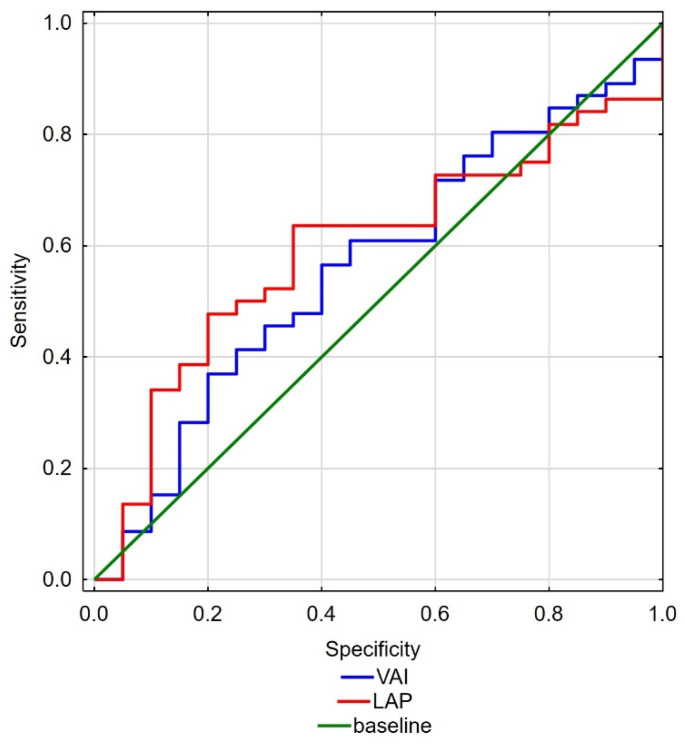
Receiver operating characteristics of the concentrations of VAI (blue line) and LAP (red line) for the International Index of Erectile Function.

ROC curves for hormone concentrations (Figure 1) and fat accumulation and activity indices (Figure 2) were also created. The cut-off point value for TT concentration in the analysis between the groups was 3.975 (AUC = 0.68; *p* = 0.0098), and the cut-off point value for SHBG concentration was 68.34 (AUC = 0.66; *p* = 0.0198). On the other hand, the cut-off point value for LAP index analysis between the groups was 63.74 (AUC = 0.64; *p* = 0.0498) and for VAI index was 3.23 (AUC = 0.63; *p* = 0.0798).

## 4. Discussion

Knowledge about the relationship between ED and metabolic disorders such as obesity and visceral fat accumulation has been developing for many years. We now have many indices that characterize the obesity problem from different perspectives based on a variety of biochemical parameters. The debate on which index is best for a simple and reliable assessment of these disorders is still ongoing. Indices such as BMI, VAI, LAP, and WHR are based on different anthropometric and biochemical parameters and cannot be treated as unequivocal. However, they may constitute a good basis for preliminary diagnosis of other disorders coexisting with excessive body weight.

A positive relationship has been shown between the degree of obesity and the incidence of inflammation. This condition is associated with a decrease in nitric oxide activity and results in endothelial damage and dysfunction, which indirectly contributes to ED. An alternative cause of ED may also be the relationship between the levels of testosterone and estradiol and obesity [17]. In our study, we have shown that there is a direct relationship between erectile dysfunction and fat accumulation indices and sex hormone levels in men over the age of 60. Indices such as VAI or LAP indicate that an excessive accumulation of visceral fat predisposes to the occurrence of ED; thus, such an assessment looks more valid than using anthropometric measurements such as BMI or WHR. The use of TG and HDL concentrations gives a more objective picture of disorders associated with abdominal obesity, in which these parameters more adequately describe the function of visceral adipose tissue, the expression of which is associated with the secretion of cytokines, adipokines, interleukin-6, tumor necrosis factor (TNF), and other proinflammatory factors. Also expressed in visceral adipose tissue is aromatase, which mediates the conversion of testosterone to estradiol [18].

The relationship between TT concentrations and ED is well established. The level of hypogonadism that induces ED is disputed and may be influenced by many factors, including extrinsic factors [19,20], patient age, and drug treatment [21]. It has been shown that low TT concentrations are directly related to higher BMI values. This is a consequence of the fact that adipose tissue is regulated by oxidative stress. This results in high concentrations of adipokines such as leptin, which inhibits testosterone secretion from Leydig cells. In addition, peripheral conversion of testosterone to estrogen is augmented, causing negative feedback inhibition on luteinizing hormone production and then inhibition of testicular androgen production [22]. Testosterone therapy can lead to improvements in erectile function, as indicated in IIEF domain scores [23]. Changes in testosterone and SHBG hormones are closely associated with aging and male sexual activity but not with sexual desire [24]. There are reports that testosterone treatment of men over 65 years of age with known low libido and decreased TT concentrations has positive effects. In this study, it was found to improve most types of sexual activity and ED and to increase both estradiol and TT concentrations. Importantly, however, we found no clinical features that predict response to TT treatment and no TT threshold for improved sexual function [25]. In our study, we demonstrated that TT and SHBG levels might also be associated with ED as factors not directly related to patient age.

Among younger men aged 20–45 years, obesity appears to be associated with erectile dysfunction. A study by Andersen et al. [26] found that the risk of erectile dysfunction in this age group is up to three times higher in those with a BMI ≥ 30 kg/m^2^ compared with a lower BMI. There are reports that WC is a good predictor of obesity-related cardiovascular risk. It has been shown by Yassin et al. [27] that in men with hypogonadism, WC is the best predictor of the quality of life in men, being inversely related to the IIEF-5 score. Body weight also correlates with the IIEF. In contrast, there is no correlation between body weight and Aging Males’ Symptoms (AMS).

The situation of patients being prepared for bariatric surgery is also worth noting. This is an extreme situation, but due to the increasing proportion of obese people in developed countries, this problem will grow and become a serious problem for health care systems. A study by Steffen et al. [28] of men and women (median BMI 46 kg/m^2^, meridian age 45 years) found that as many as a quarter of the men surveyed were sexually inactive, and as many as 12% reported a lack of sexual desire. Among the sexually active men, as many as 54% were moderately or very dissatisfied with their sex lives. Similar to our study, it has been shown that VAI may be significantly higher in ED patients. Interestingly, in the multivariate analysis, the VAI value was shown to be more strongly associated with ED than each other parameter used to calculate the index when analyzed separately. Dursun et al. [29] suggested that the reason for this condition may be that the parameters characterized by the VAI index reflect the indirect effects of other risk factors—increased adipocytokine production and elevated levels of lipolysis and free fatty acids.

Our study was associated with a number of limitations. First, it was based on information provided via questionnaires, and we had no control over the accuracy of the responses; their veracity may have been affected by various personal and social factors. Another weakness of the study was the method of hormone determination. Although ELISA is a well-established method, we are aware of the possible measurement errors of this method, especially in the determination of TT levels.

## 5. Conclusions

In our study, VAI and LAP have been shown to be good indicators for assessing erectile dysfunction in men over 60 years of age. The use of indices with the inclusion of biochemical parameters in the calculation of indices of body fat accumulation and activity allows for a more accurate assessment that also takes into account other risk factors that may affect sexual function. In addition, it is worth noting that the inclusion of biochemical assessment in the ED examination does not significantly increase the cost of diagnosis and provides an opportunity to diagnose other life-threatening diseases. Lifestyle modification and weight reduction, as well as control of lipid parameters, seem to be important in the prevention of erectile dysfunction in men. It is also worth emphasizing that the study of metabolism and adipose tissue activity may indirectly reflect changes in TT and SHBG concentrations in the male body. This research is worth developing and performing on a larger population. It may indicate the need for changes in the diagnosis of ED in patients with increased body weight, providing new and simple methods that will facilitate the initial diagnosis at the stage of contact with the general practitioner.

## Figures and Tables

**Table 1:** Analysis of the relationship between anthropometric parameters in men depending on the score obtained in the International Index of Erectile Function.

Parameters	Patients with Erectile Dysfunction *n* = 72				Patients without Erectile Dysfunction *n* = 34				*p*
	X	SD	Min	Max	X	SD	Min	Max	
Age (years)	65.48	4.28	60.00	75.00	64.68	4.52	60.00	74.00	0.382
Body weight (kg)	90.88	14.91	57.00	125.00	90.92	12.34	70.00	114.00	0.900
WC (cm)	105.60	11.50	73.00	126.00	103.10	9.54	80.00	118.00	0.352
BMI (kg/m^2^)	29.54	4.44	14.99	42.97	29.52	3.73	21.91	37.89	0.927
WHR	1.03	0.06	0.89	1.26	1.00	0.06	0.94	1.16	0.044 *
VAI	3.07	1.69	0.88	8.30	2.32	1.22	0.84	5.55	0.048 *
LAP	84.03	43.18	8.39	192.36	63.96	25.59	34.48	133.44	0.050 *
HOMA-IR	3.76	7.36	0.58	52.86	2.79	3.59	0.64	19.11	0.969

Abbreviations: X, arithmetic mean; SD, standard deviation; Min, minimum; Max, maximum; *p*, statistically significant value; *n*, number; BMI, body mass index; WC, waist circumference; WHR, waist-to-hip ratio; VAI, visceral adiposity index; LAP, lipid accumulation product; HOMA-IR, homeostatic model assessment for insulin resistance; *, statistically significant difference.

**Table 2:** Analysis of the relationship between hormonal parameters in men according to the score in the International Index of Erectile Function.

Parameters	Patients with Erectile Dysfunction *n* = 72				Patients without Erectile Dysfunction *n* = 34				*p*
	X	SD	Min	Max	X	SD	Min	Max	
TT (ng/mL)	4.79	2.12	1.38	10.54	5.75	2.29	1.69	12.90	0.018 *
FT (ng/mL)	0.09	0.04	0.02	0.23	0.08	0.04	0.02	0.16	0.501
Bioavailable T (ng/dL)	2.00	0.90	0.46	5.06	1.92	0.95	0.42	3.87	0.735
E2 (pg/mL)	82.52	35.45	28.26	180.53	89.77	38.39	20.92	170.83	0.442
SHBG (nmol/L)	45.95	29.87	5.68	143.55	75.42	57.66	12.65	215.49	0.026 *
DHEAS (µg/mL)	0.88	0.65	0.11	3.28	0.98	0.77	0.09	2.75	0.773

Abbreviations: X, arithmetic mean; SD, standard deviation; Min, minimum; Max, maximum; *p*, statistically significant value; *n*, number; TT, total testosterone; FT, free testosterone; E2, estradiol; SHBG, sex hormone-binding globulin; DHEAS, dehydroepiandrosterone sulfate; *, statistically significant difference.

**Table 3:** Analysis of the relationship between biochemical parameters in men depending on the score in the International Index of Erectile Function.

Parameters	Patients with Erectile Dysfunction *n* = 72				Patients without Erectile Dysfunction *n* = 34				*p*
	X	SD	Min	Max	X	SD	Min	Max	
ChT	180.54	49.61	105.3	308.7	179.85	47.01	97.9	264.6	0.871
HDL	39.42	9.75	18.2	63.1	41.83	9.87	22.6	56.6	0.201
TG	196.02	157.52	11.4	1033	176	105.65	77.6	583.7	0.201
LDL	113.2	82.14	36.46	612.68	103.57	45.16	12.28	189.9	0.779
FPG	86.78	29.26	59.1	134.3	80.66	18.2	58.7	128.8	0.957
Albumins (g/dL)	4.12	0.31	3.2	4.9	4.31	0.31	3.6	5.1	0.029 *

Abbreviations: X, arithmetic mean; SD, standard deviation; Min, minimum; Max, maximum; *p*, statistically significant value; *n*, number; ChT, total cholesterol; HDL, high-density lipoprotein; TG, triglycerides; LDL, low-density lipoprotein; FPG, fasting plasma glucose; *, statistically significant difference.

**Table 4:** Multivariate logistic regression explaining the International Index of Erectile Function score, adjusted for age.

Parameters	*p*	OR	Confidence OR−95%	Confidence OR 95%
VAI	0.072	1.536	0.962	2.453
LAP	0.050 *	1.017	1.000	1.034
BMI	0.604	1.035	0.908	1.181
TT	0.046 *	1.216	0.967	1.530
SHBG	0.007 *	1.020	1.005	1.036

Abbreviations: *p*, statistically significant value; OR, odds ratio; VAI, visceral adiposity index; LAP, lipid accumulation product; BMI, body mass index; TT, total testosterone; SHBG, sex hormone-binding globulin; *, statistically significant difference.

## Data Availability

The data presented in this study are available on request from the corresponding author.
